# Laser interstitial thermal therapy in pediatric neurosurgery: a prospective nationwide study on indications, safety, and early outcomes

**DOI:** 10.1007/s00381-026-07438-y

**Published:** 2026-09-02

**Authors:** Silas Haahr Nielsen, Jon Foss-Skiftesvik, Torstein Ragnar Meling, Astrid Marie Sehested, Christina Engel Høi-Hansen, Michael Thude Callesen, Gorm von Oettingen, Adam Espe Hansen, Jane Skjøth-Rasmussen, Rune Rasmussen

**Affiliations:** 1 Department of Neurosurgery, Rigshospitalet, Copenhagen, Denmark; 2 Danish Comprehensive Cancer Center Brain Tumor Center, Copenhagen, Denmark; 3 Department of Paediatrics and Adolescent Medicine, Rigshospitalet, Copenhagen, Denmark; 4 Department of Clinical Medicine, University of Copenhagen, Copenhagen, Denmark; 5 Department of Paediatrics and Adolescent Medicine, Odense University Hospital, Odense, Denmark; 6 Department of Neurosurgery, Aarhus University Hospital, Aarhus, Denmark; 7 Department of Radiology, Rigshospitalet, Copenhagen, Denmark

**Keywords:** Laser interstitial thermal therapy, Stereotactic laser ablation, Pediatric neurosurgery, Epilepsy surgery, Pediatric brain tumors, Minimally invasive

## Abstract

**Purpose:**

To evaluate indications, safety, and early outcomes of MRI-guided laser interstitial thermal therapy (LITT) in a nationwide prospective pediatric cohort and to assess the feasibility of same-session stereotactic biopsy.

**Methods:**

All consecutive pediatric patients (≤ 18 years) who underwent LITT at the Danish National Referral Center between June 2021 and October 2025 were prospectively enrolled. Demographic, clinical, radiological, and surgical variables were recorded. Safety outcomes included complications, neurological deficits, length of stay, and 30-day readmission. Seizure outcomes were assessed by Engel classification and tumor response on follow-up MRI.

**Results:**

A total of 27 children (mean age 11.6 ± 4.3 years) underwent LITT, including 18 treated for drug-resistant epilepsy and 9 for tumor indications. Same-session stereotactic biopsy was performed in 10 patients without biopsy-related complications or interference with ablation. Transient neurological deficits were observed in 5 patients and persistent deficits in 3. Hospital stay was 1–2 days in 23 patients, and no 30-day readmissions occurred. Seizure freedom (Engel class I) was achieved in 67% and 50% at 12 and 24 months, respectively. In tumor patients, clinical and MRI follow-up were available in all 9 patients, with one radiographic recurrence during follow-up.

**Conclusion:**

In this nationwide prospective cohort of 27 consecutively treated children, LITT demonstrated a favorable safety profile across epilepsy and tumor indications. Same-session stereotactic biopsy was feasible and safe, supporting LITT as a minimally invasive option in carefully selected pediatric patients.

## Introduction

MRI-guided laser interstitial thermal therapy (LITT) is a minimally invasive neurosurgical technique that enables targeted thermal ablation of intracranial lesions. By delivering controlled near-infrared energy through a stereotactically placed laser fiber with real-time temperature monitoring, LITT achieves precise tissue ablation while minimizing injury to surrounding brain structures [15, 19, 26, 35, 42]. The technique has emerged as a promising alternative to conventional open surgery for lesions located in deep or eloquent regions where resection carries substantial risk [5, 13, 17, 22, 43, 45, 49].

Since its clinical introduction in Europe in 2018, LITT has been increasingly adopted for a range of neurological conditions [32]. In adults, evidence in neuro-oncology consists of retrospective and prospective case series and supports the safety and efficacy of this technique for newly diagnosed and recurrent gliomas, as well as previously irradiated or deep-seated secondary brain tumors [28, 32]. For epilepsy, LITT achieves seizure control rates comparable to open resection in matched cohorts, with shorter hospital stays, reduced complication rates, and a lower incidence of permanent neurological deficits [37].

In pediatric neurosurgery, evidence for the use of LITT remains more limited. A recent systematic review and pooled analysis of 354 pediatric patients with drug-resistant epilepsy reported seizure freedom (Engel class I) in 57% at a mean follow-up of 16 months, with the best outcomes observed in patients with lesional epilepsy and hypothalamic hamartomas [53]. For pediatric brain tumors, conventional treatment continues to rely on open resection, which carries a risk of morbidity associated with surgical access, in particular for deep-seated or midline lesions [6, 14, 16]. The minimally invasive nature of LITT offers the potential to address selected tumors while reducing morbidity and facilitating early postoperative recovery and adjuvant therapy [7, 35, 46, 54]. Pediatric case series have demonstrated the feasibility of LITT for diverse pathologies, including low-grade gliomas (LGGs), gangliogliomas, dysembryoplastic neuroepithelial tumors (DNETs), subependymal giant cell astrocytomas (SEGAs), and ependymomas, with encouraging safety profiles [1, 27, 35, 46, 55]. Nonetheless, most available data derive from single-center, retrospective cohorts, underscoring the need for systematically collected, prospective evidence [35].

In pediatric tumor neurosurgery, obtaining a tissue diagnosis with contemporary molecular profiling is central to treatment planning, particularly for low-grade gliomas in which targeted therapies are increasingly used [40, 41]. Several pediatric LITT series have reported performing stereotactic biopsy in the same session as ablation, demonstrating technical feasibility [1, 3, 35, 36, 45]. However, these reports are limited to small, retrospective series. To our knowledge, no prospective study has systematically evaluated the safety of combining biopsy with LITT in children.

To address these gaps, we conducted a 4-year prospective cohort study of consecutively treated pediatric patients undergoing LITT at a national referral center in Denmark. The study aimed to evaluate operative safety, length of stay, and early clinical and radiographic outcomes across epilepsy and tumor indications, providing one of the first systematically collected prospective datasets in the pediatric LITT field.

## Methods

### Study design and inclusion

This prospective cohort study was approved by the National Danish Research Ethics Committee (Project ID: H-21047703). All pediatric patients (≤ 18 years) who underwent LITT at Rigshospitalet between June 1, 2021, and October 31, 2025, were consecutively enrolled.

Patients treated with LITT for epilepsy indications were evaluated and followed according to the Danish National Epilepsy Surgery Program, including a standardized preoperative workup and a 2-year postoperative follow-up. The indication for LITT in epilepsy cases was established at a multidisciplinary epilepsy surgery conference. LITT was considered primarily for hypothalamic hamartomas and for epileptogenic lesions with a well-defined target and expected complete ablation coverage, particularly when the lesion was deep-seated or located in an eloquent area where open resection was considered to carry a higher risk of neurological morbidity. In lesional cases, target selection required concordance between seizure semiology, scalp EEG findings, and structural imaging, with adjunct studies such as PET or SPECT considered when available. MRI-negative cases were considered only after invasive confirmation of the seizure-onset zone.

For oncological indications, treatment decisions were discussed at a regional or national multidisciplinary tumor board before informed consent was obtained. LITT was considered for tumors with a stereotactically accessible target when complete or near-complete ablation was considered achievable, particularly for deep-seated lesions or lesions adjacent to eloquent structures where open surgery was considered to carry a higher risk of neurological morbidity.

### Data collection

Demographic, clinical, radiological, surgical, and outcome data were prospectively obtained from electronic medical records and imaging archives. Recorded variables included sex, age at treatment, indication (epilepsy or tumor), and lesion location. Surgical parameters comprised stereotactic technique, biopsy in the same session as LITT, awake setup, number of laser fibers, diffusion tip length (3 or 10 mm), number of retractions, ablation volume including the contrast-enhancing rim (volume was calculated using the ellipsoid approximation: *V* = (*π*/6) × length × height × width) and operative time (defined as the interval from stereotactic setup to skin closure following catheter removal).

Postoperative variables included steroid and opioid use, length of stay, postoperative disposition, and 30-day readmission. Length of hospital stay was defined as the number of nights spent in hospital following the LITT procedure, with a stay of 1 day indicating discharge on postoperative day 1. Complications were categorized as none, transient neurological deficit (resolved within 30 days), or persistent deficit (present beyond 3 months). Hypothalamic hamartoma–specific sequelae (weight gain, appetite change, memory disturbance, or endocrinologic abnormalities) were also recorded.

Follow-up data included clinical and radiographic outcomes and length of follow-up. For epilepsy patients, etiology, age at seizure onset, latency to LITT, number of antiseizure medications (ASMs) at treatment, prior interventions, and Engel classification at 12 and 24 months were collected. For tumor patients, histology, recurrence, and radiographic response at last follow-up were documented. Time to last follow-up, survival status, and date of last contact were noted for all patients.

### Surgical technique

All procedures were performed using the Visualase system (Medtronic, Minneapolis, MN, USA). Laser catheter placement and, when indicated, biopsy were guided by either a frame-based stereotactic system (Cosman-Robert-Wells, Integra LifeSciences, Plainsboro, NJ, USA) or an MRI-guided SmartFrame Array (ClearPoint Neuro, Irvine, CA, USA). The SmartFrame system was preferentially used for small, deep-seated targets requiring high stereotactic accuracy, which in this case series included hypothalamic hamartomas. In frame-based cases, biopsy and laser catheter placement were performed in the operating room before transfer to the intraoperative MRI suite for thermography-guided ablation. When same-session biopsy was performed, tissue sampling and LITT were carried out through the same stereotactic trajectory. Image registration and trajectory verification were performed using an intraoperative CT scanner (AIRO, Brainlab AG, Munich, Germany). Real-time MR thermography was conducted on a 1.5-T intraoperative MRI system (GE Healthcare, Chicago, IL, USA).

### Statistical analysis

Continuous variables are reported as mean ± standard deviation (SD) or median (interquartile range (IQR)), as appropriate; categorical variables are presented as counts and percentages.

## Results

### Cohort characteristics

A total of 27 pediatric patients (mean age at LITT, 11.6 ± 4.3 years) underwent LITT between June 2021 and October 2025 (Tables 1 and 2). The cohort comprised 14 females (52%) and 13 males (48%). The primary indication was epilepsy in 18 patients (67%) and tumor in 9 patients (33%). Ablated regions included temporal, hypothalamic, callosal, parietal, frontal, intraventricular, cerebellar, occipital, thalamic, and insular locations; the callosal cases comprised one corpus callosotomy and two low-grade gliomas.

**Table 1:** Patient-level characteristics and outcomes by treatment indication (epilepsy cohort (*n* = 18))

Sex	Age, years	Pathology (etiology)	Region ablated	Ablation vol, cm^3^ (% ablated)	Adjuncts	Latest Engel	FU, months	Procedure-related morbidity
F	17	Corpus callosotomy (tuberous sclerosis)	Corpus callosum	11.00	None	IIIA	19	Persistent: mild right-sided hemiparesis
M	18	Diffuse astrocytoma (*MYB/MYBL1*)	Parietal	1.94 (100)	SEEG; Bx	IA	13	None
F	7	FCD	Fusiform gyrus	4.91 (100)	None	IA	43	None
F	17	FCD	Orbitofrontal	2.56 (100)	SEEG; Prior resection	IVB	39	None
F	16	Ganglioglioma (*BRAF*)	Temporal	2.67 (100)	Bx	IA	48	None
M	15	Ganglioglioma (*NTRK2*)	Temporal	1.36 (100)	Bx	IA	13	None
F	7	HH	Hypothalamic region	0.18 (95)	None	IA	20	None
M	8	HH	Hypothalamic region	3.06 (70)	None	IA	12	None
F	9	HH	Hypothalamic region	0.23 (100)	None	IA	13	Transient: mild right-sided hemiparesis
F	11	HH	Hypothalamic region	0.40 (100)	None	IA	15	None
F	13	HH	Hypothalamic region	0.76 (100)	None	IA	15	Persistent: memory deficit
M	2	HH (Pallister–Hall syndrome)	Hypothalamic region	2.09 (100)	None	IA	24	Transient: mild left hemiparesis
M	10	MRI-negative ETLE	Insula	1.73 (100)	SEEG	IVB	39	Transient: mild right hemiparesis
F	18	MRI-negative ETLE	Occipital	3.70 (100)	SEEG	IVB	28	Persistent: left upper partial quadrantanopia
M	8	MTS	Temporal	3.77	None	IA	14	None
F	16	MTS	Temporal	4.28	None	IA	30	None
M	12	MTS (previous leukemia)	Temporal	3.96	None	IIA	18	None
F	18	Poststroke epilepsy	Parietal	2.06 (100)	SEEG; Awake	IIIA	30	None

Includes patients treated for epilepsy. Tumor-associated epilepsy cases are listed because seizure control was the primary indication for LITT. Within each panel, rows are sorted by pathology. Ablation volume is shown as cm^3^, with percent ablated in parentheses when available

*Bx*, biopsy; *ETLE*, extratemporal lobe epilepsy; *FCD*, focal cortical dysplasia; *FU*, follow-up; *HH*, hypothalamic hamartoma; *LITT*, laser interstitial thermal therapy; *MTS*, mesial temporal sclerosis; *RT*, radiotherapy; *SEEG*, stereoelectroencephalography; *SEGA*, subependymal giant cell astrocytoma

**Table 2:** Patient-level characteristics and outcomes by treatment indication (tumor cohort (*n* = 9))

Sex	Age, years	Pathology (etiology)	Region ablated	Ablation vol, cm^3^ (% ablated)	Adjuncts	Oncologic status at last FU	FU, months	Procedure-related morbidity
M	7	Anaplastic ependymoma	Cerebellum + parieto-occipital	2.68 (95)	Bx; 4 prior resections + RT	Recurrence at 93 d; death at 334 d	11	Transient: mild dysphagia
M	12	Ganglioglioma	Amygdala	1.76 (100)	Bx	No recurrence	15	None
M	9	Glioneuronal tumor (NF-1)	Frontal	1.41 (100)	Bx	No recurrence	24	None
F	12	Low-grade glioma (*NF-1*)	Corpus callosum	1.04 (100)	Bx	No recurrence	7	None
M	6	Low-grade glioma (*NF-1*)	Thalamus	1.95 (100)	Bx	No recurrence	9	None
F	13	Pilocytic astrocytoma (*NF-1*)	Corpus callosum	2.70 (100)	Bx; prior biopsy	No recurrence	7	None
F	13	Pilocytic astrocytoma (*NF-1*)	Right cerebellar peduncle	2.35 (100)	Bx; Vinblastine	No recurrence	8	Transient: mild balance difficulty
M	8	SEGA (tuberous sclerosis)	Intraventricular	0.90 (100)	None	No recurrence	18	None
M	12	SEGA (tuberous sclerosis)	Intraventricular	0.85 (100)	None	No recurrence	19	None

Includes patients treated for tumors. Within each panel, rows are sorted by pathology. Ablation volume is shown as cm^3^, with percent ablated in parentheses when available

*Bx*, biopsy; *ETLE*, extratemporal lobe epilepsy; *FCD*, focal cortical dysplasia; *FU*, follow-up; *HH*, hypothalamic hamartoma; *LITT*, laser interstitial thermal therapy; *MTS*, mesial temporal sclerosis; *RT*, radiotherapy; *SEEG*, stereoelectroencephalography; *SEGA*, subependymal giant cell astrocytoma

### Procedural characteristics

A frame-based stereotactic system was used in 23 cases (85%), while the MRI-guided SmartFrame was applied in 4 cases (15%) [39], the latter targeting hypothalamic hamartomas. Same-session biopsy was performed in 10 patients, including 7 with tumor indications (excluding subependymal giant cell astrocytomas [SEGAs]) and 3 patients with epilepsy in whom a low-grade glioma or glioneuronal tumor was suspected. In all biopsy cases, tissue sampling and LITT were performed through the same trajectory. There were no biopsy-related complications, and the combined single-session biopsy-LITT strategy did not compromise the subsequent ablation, defined as no bleeding or air artifact interference.

One patient underwent awake LITT with intraoperative language testing; the ablation was tailored to reduce the risk of permanent postoperative aphasia [34]. The procedure included preoperative constrained spherical deconvolution (CSD) tractography performed with Quicktome software (Omniscient Neurotechnology) and intraoperative language assessment after a controlled temperature increase to approximately 46 °C for less than 30 s.

A single laser fiber was used in 25 patients (93%), two fibers in 1 patient (4%), and three fibers in 1 patient (4%). The diffusion-tip length was 10 mm in 26 cases and 3 mm in 1 case. The median number of retractions was 2 (IQR, 1–2.5). Mean ablation volume was 2.5 ± 2.1 cm^3^. Mean operative time was 170.8 ± 62.5 min. When using the MRI SmartFrame, mean operative time was 270.0 ± 55.8 min, compared with 153.5 ± 45.7 min when using the frame-based stereotactic system.

### Complications and postoperative course

No catheter repositioning, hemorrhage, or anesthesia-related complications occurred. Postoperatively, 19 patients (70%) had no new neurological deficits. New neurological deficits occurred in 8 patients (30%), of whom 5 (19%) were transient (resolved within 30 days) and 3 (11%) were persistent (present beyond 3 months). Transient deficits included mild motor deficits after three epilepsy procedures: two hypothalamic hamartoma ablations, in which the trajectory passed through the primary motor cortex to achieve optimal ablation coverage, and one insular MRI-negative epilepsy ablation associated with edema in close proximity to the corticospinal tract. Other transient deficits included mild dysphagia after LITT for recurrent ependymoma in the posterior fossa/fourth ventricle and mild balance disturbance after cerebellar pilocytic astrocytoma ablation. Persistent deficits occurred in 3 patients: left upper partial quadrantanopia after occipital MRI-negative epilepsy ablation, persistent right-sided mild hemiparesis after corpus callosotomy due to slight superior misplacement of one of three catheters with related edema, and memory impairment after hypothalamic hamartoma ablation with thermal damage involving the fornices.

Opioid administration for postoperative pain was required in one patient (4%). Corticosteroids were administered in 13 patients (48%), most commonly for ≤ 3 days. The length of hospital stay was 1 day (discharge on postoperative day 1) in 18 patients (67%), 2 days in 5 (19%) and ≥ 3 days in 4 (15%). There were no postoperative infections or readmissions within 30 days.

**Fig. 1:**
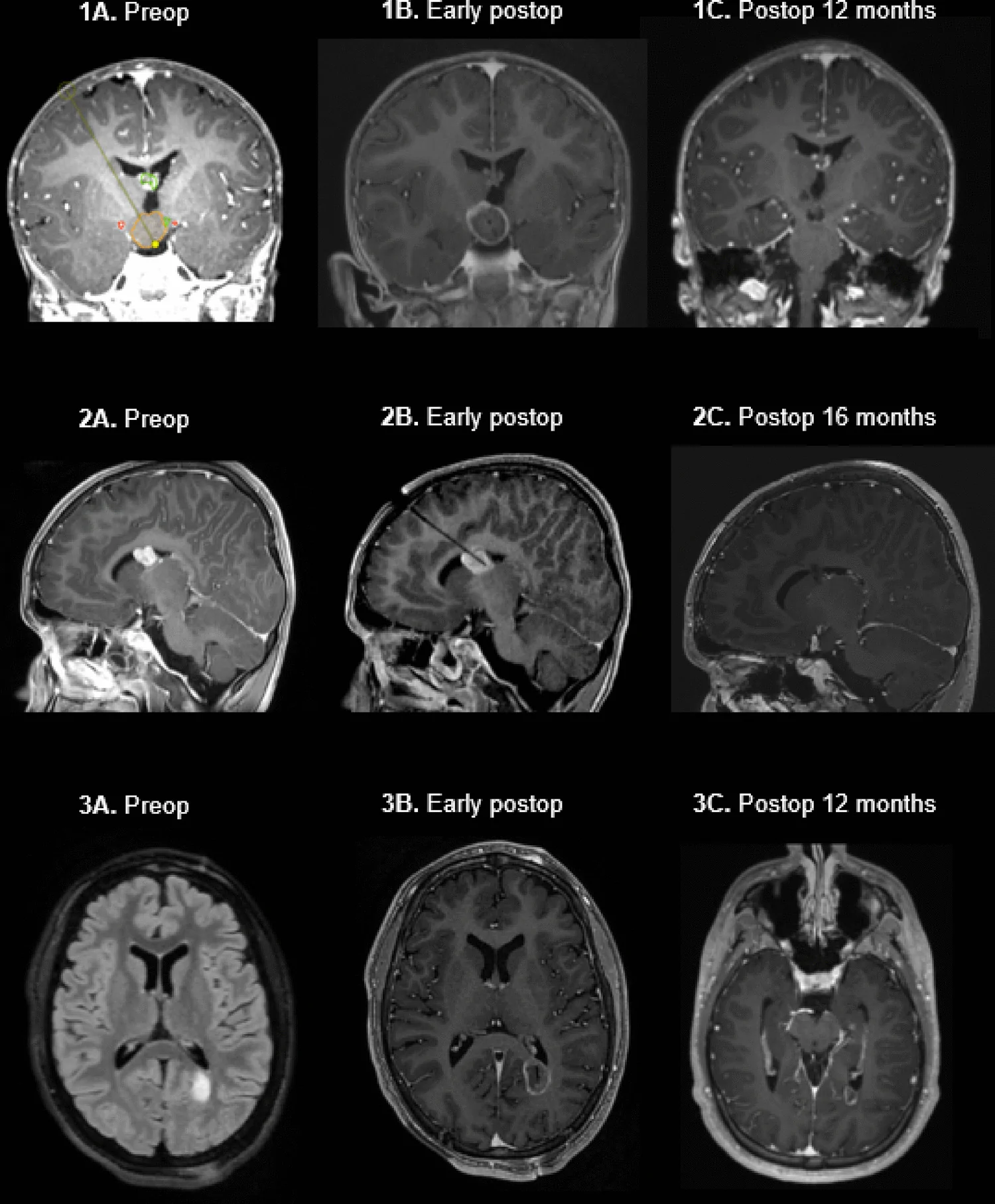
Illustrative pediatric LITT cases. Preoperative MRI (**A**), early postoperative MRI (**B**), and follow-up MRI (**C**) showing posttreatment changes in hypothalamic hamartoma (1), subependymal giant cell astrocytoma (2), and pilocytic astrocytoma (3), with follow-up MRI obtained at 12 months, 16 months, and 12 months for cases 1, 2, and 3, respectively

Among the children with hypothalamic hamartoma (*n* = 6, Fig. 1, panels 1A–C), all had pre- and postoperative endocrinological workup and neuropsychological assessment. Two experienced transient postoperative appetite changes (one increased, one decreased). One child was evaluated postoperatively for suspected central precocious puberty and was subsequently found to have developed postoperative hypothyroidism requiring treatment. No other persistent endocrinological deficits were observed.

In four children, neither patient- nor parent-reported assessments revealed postoperative memory complaints, except for cognitive fatigability. One child demonstrated a possible subtle decline in verbal memory despite improvement on broader cognitive testing. The final patient developed marked postoperative memory impairment, consistent with injury to the fornices. No other new neuropsychiatric findings were observed.

### Epilepsy outcomes

Among the 18 patients treated for epilepsy, pathologies included hypothalamic hamartoma (*n* = 6), mesial temporal sclerosis (*n* = 3), extratemporal MRI-negative epilepsy (*n* = 2), focal cortical dysplasia (*n* = 2), ganglioglioma (*n* = 2), diffuse astrocytoma (*n* = 1, Fig.  1, panels 3A–C), poststroke epilepsy (*n* = 1), and tuberous sclerosis with drug-resistant atonic seizures managed with corpus callosotomy-assisted LITT (*n* = 1). The mean age at seizure onset was 5.2 ± 4.8 years, with a mean interval of 7.2 ± 4.7 years from epilepsy onset to LITT. The median number of antiseizure medications at treatment was 2 [1-2]. The two patients not receiving antiseizure medication (ASM) at the time of LITT both had hypothalamic hamartoma. Prior interventions included resective surgery in one patient (6%), in whom LITT was used to target residual orbitofrontal dysplasia. Five patients (28%) underwent SEEG before LITT, including both MRI-negative cases and three lesional cases.

Seizure freedom (Engel class I) was achieved in 67% of patients at 1 year and 50% at 2 years (Tables 3, 4, and 5). In total, 13 of 16 patients (81%) with 1-year follow-up had seizure reduction, whereas 3 of 16 (19%) had no appreciable change. Among patients with MRI-negative epilepsy, neither of the two patients was seizure free at 1 year, compared with 71% (10/14) among the remaining epilepsy patients with 1-year follow-up. The median follow-up duration was 19.5 (14.0–30.0) months.

**Table 3:** Epilepsy cohort: indications and seizure outcomes (seizure freedom by indication)

Indication	***n***	Seizure-free at 1 year	%
Hypothalamic hamartoma	6	6/6	100
Mesial temporal sclerosis	3	2/3	67
Extratemporal MRI-negative	2	0/2	0
Dysplasia	2	1/2	50
Ganglioglioma	2	2/2	100
Diffuse astrocytoma	1	1/1	100
Corpus callosotomy	1	0/1	0
Post-stroke epilepsy	1	0/1	0
Total	18	12/18	67

Values are *n* (%)

**Table 4:** Epilepsy cohort: indications and seizure outcomes (seizure outcome by Engel classification)

Engel class^a^	1 year (*n* = 18)	2 years (*n* = 8)
I—seizure freedom	12 (67%)	4 (50%)
II—rare disabling seizures	1 (6%)	0
III—worthwhile improvement	2 (11%)	1 (13%)
IV—no worthwhile improvement	3 (17%)	3 (38%)

^a^Engel I, seizure freedom or auras only; II, rare disabling seizures; III, worthwhile improvement; IV, no worthwhile improvement. *N* reflects patients with available follow-up data at each timepoint

**Table 5:** Epilepsy cohort: indications and seizure outcomes (clinical parameters)

Variable	Value
Follow-up (months, median [IQR])	19.5 [14.0–30.0]
Age at epilepsy onset (years, mean ± SD)	5.2 ± 4.8
Time from onset to LITT (years, mean ± SD)	7.2 ± 4.7
ASMs at time of LITT (median [IQR])	2 [1-2]
Prior interventions	
None	12
Resection	1
SEEG	5

Values are *n* or summary statistics as indicated.

*ASM*, antiseizure medication; *IQR*, interquartile range; *SD*, standard deviation; *SEEG*, stereoelectroencephalography

### Tumor outcomes

Nine patients underwent LITT for tumor indications (Table 6), including low-grade glioma in patients with neurofibromatosis type 1 (*n* = 4), subependymal giant cell astrocytoma in patients with tuberous sclerosis (SEGA; *n* = 2, Fig. 1.2A–C), glioneuronal tumor (*n* = 2), and ependymoma (*n* = 1). Treated tumor locations included the frontal lobe, amygdala, thalamus, corpus callosum, intraventricular region, cerebellum, and parieto-occipital/posterior fossa. The median follow-up duration was 11 [7.5–18.5] months. Clinical and MRI follow-up were available in all 9 patients. One patient developed tumor recurrence during follow-up. This patient had undergone four prior resections and radiation therapy for a recurrent anaplastic posterior fossa type A ependymoma involving the parieto-occipital region and cerebellum/fourth ventricle and was treated with LITT using two catheters. Tumor recurrence occurred 93 days after LITT, and the patient died 334 days post-procedure. The remaining eight patients had no radiographic recurrence at last follow-up.

**Table 6:** Tumor cohort: indications and follow-up outcomes

Variable	Value
Indication	
Low-grade glioma	4
SEGA	2
Glioneuronal tumor	2
Ependymoma	1
Length of follow-up (months, median [IQR])	11 [7.5–18.5]
Follow-up MRI available	9/9
Tumor recurrence	1/9
Tumor control at last follow-up	8/9

Values are *n* or summary statistics as indicated

Low-grade glioma includes pilocytic astrocytoma (*n* = 2) and low-grade glioma (*n* = 2). Glioneuronal tumor includes ganglioglioma (*n* = 1) and glioneuronal tumor (*n* = 1)

*FU*, follow-up; *IQR*, interquartile range; *SEGA*, subependymal giant cell astrocytoma

## Discussion

This prospective single-center cohort addresses an important evidence gap in pediatric neurosurgery by providing prospectively collected data on the safety and early outcomes of LITT across both epilepsy and tumor indications. Among 27 consecutively treated children, the rate of persistent neurological morbidity was low. Same-session stereotactic biopsy was performed in ten patients and did not compromise the subsequent ablation. Overall, encouraging early outcomes were observed in both subgroups: seizure control was achieved in the majority of epilepsy patients, and no recurrences were observed among the low-grade tumors during the available follow-up. Taken together, these findings support LITT as a safe and feasible minimally invasive option in selected pediatric patients, while underscoring that outcomes are highly dependent on indication, target definition, and patient selection.

### Role of LITT in pediatric epilepsy surgery

In the epilepsy subgroup, seizure outcomes in the present cohort are broadly comparable to those reported in the largest retrospective pediatric LITT series and pooled analyses, which describe Engel class I rates of approximately 57%, with the most favorable results observed in lesional epilepsies and hypothalamic hamartomas [37, 53, 55]. Our prospective study confirms this pattern, with excellent seizure control in hypothalamic hamartomas and other well-defined lesions, and less favorable outcomes in MRI-negative epilepsies. In our practice, the most suitable epilepsy candidates for LITT have been children with hypothalamic hamartoma or with a well-defined epileptogenic lesion in which complete or near-complete ablation coverage is expected, particularly when the target is deep-seated or situated in an eloquent area where open resection is considered to carry higher access-related neurological risk. By contrast, MRI-negative cases were considered only after invasive confirmation and still yielded less favorable outcomes in this series. When compared with conventional resective epilepsy surgery, which can achieve seizure freedom in up to two-thirds of carefully selected children with focal lesional epilepsy at 2-year follow-up [12], LITT appears to offer a minimally invasive alternative in selected cases, but its effectiveness remains highly dependent on accurate lesion localization, concordant presurgical data, and careful target definition [1, 15, 25, 37, 44, 53].

### Role of LITT in pediatric neuro-oncology

In the tumor cohort, clinical and radiographic follow-up were available in all patients, and 8 of 9 patients had no recurrence at last follow-up. The only recurrence occurred in a patient with recurrent multifocal anaplastic posterior fossa type A ependymoma, a finding that likely reflects the aggressive biological behavior of this entity rather than a technical limitation of the ablation, as further cytoreduction was unlikely to alter the natural disease course. In our practice, the most suitable tumor candidates for LITT have been children with growing low-grade lesions in which complete or near-complete ablation is considered achievable, particularly when the target is deep-seated or adjacent to eloquent structures and an open approach is considered to carry a higher risk of neurological morbidity. Our findings are consistent with prior retrospective studies reporting LITT primarily for low-grade gliomas, gangliogliomas, SEGAs, and occasional ependymomas in deep or surgically complex locations, including the mediotemporobasal region, insula, cingulate gyrus, basal ganglia, hypothalamus, brainstem, and cerebellum [1, 3, 9, 27, 35, 36, 44, 49]. An important practical advantage in our series was the ability to combine diagnostic biopsy and therapeutic ablation within a single operative session, thereby potentially reducing the need for repeated anesthesia exposure while obtaining tissue for histopathological and molecular characterization. This approach was particularly relevant for children with known NF1 undergoing routine radiological surveillance, in whom incidental slow-growing lesions without prior histological diagnosis could be simultaneously biopsied and ablated. Targeted treatments are now available for a broad spectrum of low-grade gliomas [8, 38]; however, in cases of treatment failure, progression, or diagnostic uncertainty, the combination of biopsy and LITT offers a minimally invasive alternative that preserves future treatment options.

### Advantages and limitations of LITT compared with other treatment modalities

Compared with open surgery, LITT may offer lower access-related morbidity, shorter hospital stays, and faster recovery, but with inherent limitations in the achievable extent of ablation [20, 30, 44–46, 48, 55]. These advantages are particularly relevant for small, deep-seated lesions or lesions in eloquent locations [17, 35].

Compared with SEEG-guided radiofrequency ablation, which is attractive for highly focal cortical lesions such as periventricular heterotopias but is inherently limited by small ablation volumes and the absence of real-time visualization of thermal spread, LITT provides continuous MR thermometry, more predictable thermal injury, and the capacity to create substantially larger ablative volumes [2, 4, 11, 24, 50, 51].

In relation to radiosurgery or Gamma Knife, LITT confers three key advantages: first, histological and molecular diagnosis can be obtained in the same session through stereotactic biopsy, enabling tailored oncologic management; second, the ablative effect is immediate, and third, the ablated lesion can be re-treated or subsequently resected if progression occurs, without the cumulative dose limitations inherent to radiation [10, 18, 21, 29, 42, 52]. For selected pediatric patients, especially those with deep low-grade tumors, LITT therefore represents a pragmatic compromise between oncological control, access to tissue, and minimization of long-term treatment-related toxicity.

### Strengths and limitations

Key strengths of this study include its national, prospective design, which ensured complete case inclusion and minimized selection bias by consecutively capturing all pediatric LITT procedures performed in the country, independent of outcomes. The use of a standardized data collection framework enabled consistent recording of demographic, clinical, radiological, technical, and outcome variables. All procedures were performed at a single high-volume national referral center, providing uniformity in surgical technique and perioperative care. However, several limitations of this study must be acknowledged. The heterogeneity of the cohort, including epilepsy and tumor indications across varied lesion locations, reflects real-world practice but limits subgroup analyses and indication-specific conclusions. The sample size is modest and the follow-up is relatively short, particularly for assessing medium- and long-term seizure recurrence, tumor progression, and neurocognitive trajectories. The present data are therefore best interpreted as early prospective benchmarks rather than definitive estimates of long-term effectiveness. In some cases, LITT was offered to children with complex or previously extensively treated disease, often when conventional options were exhausted or associated with substantial risk, which may bias outcomes downward relative to first-line use in more straightforward lesions. Our findings also derive from a single national center during the early phase of LITT implementation, which may limit generalizability to units with different case mixes and technologies. Finally, in the absence of a matched control group treated with resection, radiosurgery, or SEEG-guided ablation, we cannot draw definitive comparative effectiveness conclusions.

### Future perspectives

Future work in this area should aim to clarify not only the clinical indications but also the biological effects of LITT. Experimental and early clinical data indicate that thermal ablation transiently disrupts the blood–brain barrier, which may enhance penetration of systemic agents into the perilesional zone and provide a therapeutic window for early adjuvant chemotherapy, targeted therapy, or immunotherapy [33]. Thermal injury may also modulate the local immune microenvironment by releasing tumor antigens and danger-associated molecular patterns, potentially augmenting anti-tumor immune responses [23, 31, 47]. These effects are particularly relevant in pediatric neuro-oncology, where minimizing radiation exposure and leveraging targeted or immune-based treatments are central goals. In parallel, multicenter studies have already been established in northern Europe for pediatric low-grade glioma, periventricular heterotopia, and hypothalamic hamartoma. Together with prospective registries, these efforts will be essential to better define patient selection, optimize the timing and sequencing of systemic therapies relative to LITT, and standardize ablation parameters and imaging endpoints. Future studies should also determine whether blood–brain barrier- or immune-modulating effects translate into improved long-term disease control.

## Conclusion

In this nationwide prospective cohort of 27 consecutively treated children, LITT was associated with a favorable safety profile for both epilepsy and tumor indications, characterized by low rates of persistent morbidity and short hospital stays. Same-session biopsy through the ablation trajectory was feasible and did not appear to compromise ablation. In selected epilepsy cases, particularly hypothalamic hamartomas and well-defined lesional targets, and in selected low-grade tumors, the early outcomes support LITT as a useful minimally invasive alternative when open surgery carries elevated risk. These findings provide practical early benchmarks for pediatric LITT implementation but also underscore the importance of careful patient selection and larger multicenter studies with longer follow-up.

## Data Availability

The datasets generated and/or analyzed during the current study are not publicly available due to patient confidentiality but are available from the corresponding author on reasonable request, subject to institutional and ethical approval.

## References

[1] Aboubakr O, Guida L, Dangouloff Ros V et al (2025) Laser interstitial thermal therapy (LITT) in pediatric neurosurgery: single center retrospective analysis of 41 consecutive procedures. Neurochirurgie (Paris) 71(6):101719 10.1016/j.neuchi.2025.101719 40915608

[2] Ahmed S, Nadeem ZA, Kamran U, Ashfaq H, Ashraf H, Ashraf M, Agarwal A, Farooq M (2024) Magnetic resonance-guided laser interstitial thermal therapy in the management of hypothalamic hamartomas: a systematic review and meta-analysis. World Neurosurg 190:463-469.e6 10.1016/j.wneu.2024.08.009 39122113

[3] Arocho-Quinones EV, Lew SM, Handler MH et al (2020) Magnetic resonance–guided stereotactic laser ablation therapy for the treatment of pediatric brain tumors: a multiinstitutional retrospective study. J Neurosurg Pediatr 26(1):13–21 10.3171/2020.1.PEDS19496 PMC7885863 32217793

[4] Banerjee C, Snelling B, Berger MH, Shah A, Ivan ME, Komotar RJ (2015) The role of magnetic resonance-guided laser ablation in neurooncology. Br J Neurosurg 29(2):192–196 10.3109/02688697.2014.996527 25665599

[5] Barnett GH, Voigt JD, Alhuwalia MS (2016) A systematic review and meta-analysis of studies examining the use of brain laser interstitial thermal therapy versus craniotomy for the treatment of high-grade tumors in or near areas of eloquence: an examination of the extent of resection and major complication rates associated with each type of surgery. Stereotact Funct Neurosurg 94(3):164–173 10.1159/000446247 27322392

[6] Campbell E, Todd L, Amato-Watkins A, O’Kane R, Sangra M, Canty M (2025) Prospective review of 30-day morbidity and mortality following surgery for brain tumours in children. Childs Nerv Syst 41(1):162 10.1007/s00381-025-06817-1 40246770

[7] Campian JL, Le SB, Ghiaseddin A et al (2026) Laser interstitial thermal therapy and adjuvant pembrolizumab in recurrent high-grade astrocytoma: a phase 1/randomized phase 2b trial. Nat Commun 17(1):1763 10.1038/s41467-026-69522-w PMC12946167 41748622

[8] Frassanito P, Thomale UW, Obersnel M, Romano A, Leblond P, Knerlich-Lukoschus F, Due-Tønnessen BJ, Thompson D, Di Rocco F, the CPN Lyon 2024 Consensus Conference Group (2025) The state of targeted therapeutic pharmacological approaches in pediatric neurosurgery: report from the European Society for Pediatric Neurosurgery (ESPN) Consensus Conference 2024. Childs Nerv Syst 41(1):149 10.1007/s00381-025-06799-0 PMC11965156 40175630

[9] Giuseppe M, Domenico C, Giulia M, Nicola O, Eugenio C, Giuseppe C (2026) Single-center longitudinal experience with MRgLITT in pediatric neurosurgery: technical evolution, expanding indications, and advanced MRI-driven planning. Childs Nerv Syst 42(1):280 10.1007/s00381-026-07381-y 42397424

[10] Guadix SW, Pandey A, Gundlach C, Walsh M, Moss NS, Souweidane MM (2024) Laser interstitial thermal therapy as a radiation-sparing approach for central nervous system tumors in children with cancer predisposition syndromes: report of a child with Li-Fraumeni syndrome. Illustrative case. J Neurosurg Case Lessons. 10.3171/CASE23595 PMC10849145 38315990

[11] Gupta K, Cabaniss B, Kheder A et al (2020) Stereotactic MRI-guided laser interstitial thermal therapy for extra-temporal lobe epilepsy. Epilepsia 61(8):1723–1734 10.1111/epi.16614 PMC8019400 32777090

[12] Harris WB, Brunette-Clement T, Wang A, Phillips HW, von Der Brelie C, Weil AG, Fallah A (2022) Long-term outcomes of pediatric epilepsy surgery: individual participant data and study level meta-analyses. Seizure 101:227–236 10.1016/j.seizure.2022.08.010 36108556

[13] Haskell-Mendoza AP, Gonzalez AT, Reason EH, Flusche AM, Chongsathidkiet P, Wachsmuth LP, Goodwin CR, Fecci PE (2025) The LITT fit in neuro-oncology: indications, imaging, and adjunctive therapies. J Neurooncol 172(1):1–11 10.1007/s11060-024-04894-x 39585599

[14] Hatoum R, Chen J-S, Lavergne P et al (2022) Extent of tumor resection and survival in pediatric patients with high-grade gliomas. JAMA Netw Open 5(8):e2226551 10.1001/jamanetworkopen.2022.26551 PMC9382445 35972743

[15] Hect JL, Alattar AA, Harford EE, Reecher H, Fernandes DT, Esplin N, McDowell M, Abel TJ (2022) Stereotactic laser interstitial thermal therapy for the treatment of pediatric drug-resistant epilepsy: indications, techniques, and safety. Childs Nerv Syst 38(5):961–970 10.1007/s00381-022-05491-x 35274185

[16] Henriksen KA, Brix N, Jakubauskaite R, Oettingen GV, Rathe M, Skjøth-Rasmussen J, Foss-Skiftesvik J, Mathiasen R (2023) Thirty-day surgical morbidity and risk factors in pediatric brain tumor surgery: a 10-year nationwide retrospective study. J Neurosurg Pediatr. 10.3171/2023.9.PEDS23351 37976503

[17] Ho WS, de Groot J, Rauschecker AM, Mueller S, Shatara MS (2025) Laser interstitial thermal therapy (LITT) for pediatric brain tumors. A review. Neuro-Oncology Pediatrics 1(1):wuaf004. 10.1093/neuped/wuaf004

[18] Jaffee S, Remick M, Tang OY, Harford EE, Gonzalez-Martinez J, Al-Ramadhani R, Welch WP, Abel TJ (2025) Outcomes of resective epilepsy surgery after focal stereotactic MR-guided laser interstitial thermal therapy for pediatric focal epilepsy: a case series. J Neurosurg Pediatr 36(3):304–311 10.3171/2025.2.PEDS24485 40479829

[19] Jethwa PR, Barrese JC, Gowda A, Shetty A, Danish SF (2012) Magnetic resonance thermometry-guided laser-induced thermal therapy for intracranial neoplasms: initial experience. Operative Neurosurgery. 71(1):133–45 10.1227/NEU.0b013e31826101d4 22653396

[20] Kaes M, Rondinelli V, Krieg SM, Jakobs M (2025) The LITTability study - evaluation of the applicability of LITT in a real-world cohort of glioma patients. Neurosurg Rev 48(1):477 10.1007/s10143-025-03644-5 PMC12130115 40457101

[21] Kondapavulur S, Bonnin DA, Perez S, Wu J, Perry A, Mueller S, Sun PP, Ho WS (2026) Laser insterstitial thermal therapy followed by craniotomy for tumor resection for pediatric diffuse midline glioma: illustrative case. J Neurosurg Case Lessons 11(14):CASE251030 10.3171/CASE251030 PMC13051672 41941828

[22] Kuo C-H, Feroze AH, Poliachik SL, Hauptman JS, Novotny EJ, Ojemann JG (2019) Laser ablation therapy for pediatric patients with intracranial lesions in eloquent areas. World Neurosurg 121:e191–e199 10.1016/j.wneu.2018.09.074 30261370

[23] Lerner EC, Edwards RM, Wilkinson DS, Fecci PE (2022) Laser ablation: heating up the anti-tumor response in the intracranial compartment. Adv Drug Deliv Rev 185:114311 10.1016/j.addr.2022.114311 PMC10589123 35489652

[24] Li Y, Gao J, Ye Z, Mu J (2023) Magnetic resonance-guided laser interstitial thermal therapy vs. stereoelectroencephalography-guided radiofrequency thermocoagulation in epilepsy patients with focal cortical dysplasia: a systematic review and meta-analysis. Front Neurol 14:1241763 10.3389/fneur.2023.1241763 PMC10625445 37928136

[25] Maroufi SF, Fallahi MS, Amirkhani N, Dehghani Arani P, Theodore JN, Cohen-Gadol AA, Sheehan JP, Van Gompel JJ (2026) Laser interstitial thermal therapy versus open resective surgery for nontumoral epilepsy: systematic review and meta-analysis of comparative studies. J Neurosurg 144(4):787–798 10.3171/2025.8.JNS25496 41576371

[26] McNichols RJ, Gowda A, Kangasniemi M, Bankson JA, Price RE, Hazle JD (2004) MR thermometry-based feedback control of laser interstitial thermal therapy at 980 nm. Lasers Surg Med 34(1):48–55 10.1002/lsm.10243 14755424

[27] Mirone G, Picariello S, Russo C, Cicala D, Spennato P, Onorini N, Quaglietta L, Covelli E, Cinalli G (2026) Magnetic resonance guided laser interstitial thermal therapy in pediatric brain tumors: an institutional case series. J Neurosurg Sci 70(1):36–46 10.23736/S0390-5616.24.06381-1 40047450

[28] Mortezaei A, Al-Saidi N, Taghlabi KM et al (2025) Laser interstitial thermal therapy for high-grade glioma: a systematic review, meta-analysis, and meta-regression. Neurosurg Focus 59(2):E10 10.3171/2025.5.FOCUS25316 40749231

[29] Muir M, Traylor JI, Gadot R, Patel R, Prabhu SS (2022) Repeat laser interstitial thermal therapy for recurrent primary and metastatic intracranial tumors. Surg Neurol Int 13:311 10.25259/SNI_418_2022 PMC9345120 35928321

[30] Mustafa M, Zaben M (2022) Minimal invasive brain surgery for epilepsy; can it be the future? J Neurol 269(11):6178–6180 10.1007/s00415-022-11360-z PMC9553844 36098841

[31] Nielsen SH, Artzi SB, Skjøth-Rasmussen J, Larsen CC, Hammouda NM, Larsen KB, Rasmussen R, Hansen AE, Kristensen BW (2026) MR-guided laser interstitial thermal therapy for recurrent glioblastoma: a case report with novel insights into histopathological changes and immunological responses. Neuropathol Appl Neurobiol 52(2):e70071 10.1111/nan.70071 PMC13035459 41912359

[32] Nielsen SH, Rasmussen R (2024) MR-guided laser interstitial thermal therapy in the treatment of brain tumors and epilepsy. Acta Neurochir 166(1):344 10.1007/s00701-024-06238-0 39167226

[33] Nielsen SH, Skjøth-Rasmussen J, Larsen VA, Carlsen JF, Larsson HBW, Christoffersen C, Rasmussen R, Hansen AE (2025) Blood-brain barrier disruption following MR-guided laser interstitial thermal therapy. Neuro-oncol Adv. 10.1093/noajnl/vdaf148 PMC12290448 40718643

[34] Nielsen SH, Skjøth-Rasmussen J, Moldrup SD, Engelmann CM, Jespersen B, Rasmussen R (2023) Awake laser ablation with continuous neuropsychological testing during treatment of brain tumors and epilepsy. Neurosurg Clin N Am 34(2):239–245 10.1016/j.nec.2022.11.003 36906330

[35] O’Leary S, Haider MA, Truong N et al (2025) Stereotactic laser ablation for pediatric central nervous system tumors: a systematic review and meta-analysis of the literature. J Neurosurg Pediatr 36(2):230–243 10.3171/2025.1.PEDS24387 40344762

[36] Pehlivan KC, Khanna PC, Elster JD, Paul MR, Levy ML, Crawford JR, Gonda DD (2021) Clinical and neuroimaging features of magnetic resonance−guided stereotactic laser ablation for newly diagnosed and recurrent pediatric brain tumors: a single institutional series. World Neurosurg 150:e378–e387 10.1016/j.wneu.2021.03.027 33722713

[37] Pichardo-Rojas D, Espinosa-Cantú CB, Valenzuela-Rangel AF, Choque-Ayala LC, Barrón-Lomelí A, Gutierrez-Herrera EA, Mejía Pérez SI, Pichardo-Rojas PS, Milanese V, Rangel-Castilla L (2025) A comparative assessment of laser interstitial thermal therapy and open resective surgery for drug-resistant epilepsy: a meta-analysis of 3873 patients. J Neurosurg 144(1):35–54. 10.3171/2025.4.JNS25386 40911916

[38] Plant-Fox AS, Tabori U (2024) Future perspective of targeted treatments in pediatric low-grade glioma (pLGG): the evolution of standard-of-care and challenges of a new era. Childs Nerv Syst 40(10):3291–3299 10.1007/s00381-024-06504-7 39085626

[39] Rasmussen R, Nielsen SH (2024) How I do it: MRI-guided stereotactic navigation for laser interstitial thermal therapy. Acta Neurochir (Wien) 166(1):444 10.1007/s00701-024-06335-0 39509068

[40] Roka K, Scheinemann K, Avula S, Maduro JH, Thomale UW, Sehested A, van Meeteren AYNS (2024) European standard clinical practice recommendations for primary pediatric low-grade gliomas. EJC Paediatr Oncol. 10.1016/j.ejcped.2024.100169

[41] Ryall S, Tabori U, Hawkins C (2020) Pediatric low-grade glioma in the era of molecular diagnostics. Acta Neuropathol Commun 8(1):30 10.1186/s40478-020-00902-z PMC7066826 32164789

[42] Salem U, Kumar VA, Madewell JE, Schomer DF, De Almeida Bastos DC, Zinn PO, Weinberg JS, Rao G, Prabhu SS, Colen RR (2019) Neurosurgical applications of MRI guided laser interstitial thermal therapy (LITT). Cancer Imaging 19(1):65 10.1186/s40644-019-0250-4 PMC6792239 31615562

[43] Schupper AJ, Chanenchuk T, Racanelli A, Price G, Hadjipanayis CG (2022) Laser hyperthermia: past, present, and future. Neuro Oncol 24(Suppl 6):S42–S51 10.1093/neuonc/noac208 PMC9629480 36322099

[44] Seaton MP, Ghauri MS, Forseth KJ, Schmidt JC, Sahyouni R, Ravindra VM, Gonda DD (2026) MRI-guided laser ablation for pediatric intracranial pathology: single center experience. Childs Nerv Syst 42(1):44 10.1007/s00381-026-07133-y PMC12830497 41577840

[45] Spacca B, Di Maurizio M, Grandoni M, Tempesti S, Genitori L (2023) Laser interstitial thermal therapy (LITT) for pediatric patients affected by intracranial tumors. Front Neurol 14:1120286 10.3389/fneur.2023.1120286 PMC10157164 37153686

[46] Strauss I, Gabay S, Roth J (2024) Laser interstitial thermal therapy (LITT) for pediatric low-grade glioma—case presentations and lessons learned. Childs Nerv Syst 40(10):3119–27. 10.21203/rs.3.rs-4249271/v1 PMC11511763 38703238

[47] Vargas LO, Himic V, Otaner F et al (2025) Modulating the glioma microenvironment with laser interstitial thermal therapy: mechanisms and therapeutic implications. J Neurooncol 176(1):99 10.1007/s11060-025-05305-5 PMC12660436 41307758

[48] Vetkas A, Germann J, Boutet A et al (2023) Laser interstitial thermal therapy for the treatment of insular lesions: a systematic review. Front Neurol. 10.3389/fneur.2022.1024075 PMC9845884 36686528

[49] Vitulli F, Tortora D, Pacetti M et al (2025) Magnetic resonance-guided laser interstitial thermal therapy (MR-gLiTT) in pediatric neurosurgery: italian perspective and literature review. Neurol Sci 46(8):3965–3972 10.1007/s10072-025-08213-8 40327178

[50] Wang Y, Xu J, Liu T, Chen F, Chen S, Xie Z, Fang T, Liang S (2020) Magnetic resonance–guided laser interstitial thermal therapy versus stereoelectroencephalography-guided radiofrequency thermocoagulation for drug-resistant epilepsy: a systematic review and meta-analysis. Epilepsy Res 166:106397 10.1016/j.eplepsyres.2020.106397 32590289

[51] Wang M, Zhou Y, Zhang Y, Shi W, Zhou S, Wang Y, Li H, Zhao R (2020) One-stage high-density focal stereo-array SEEG-guided radiofrequency thermocoagulation for the treatment of pediatric giant hypothalamic hamartomas. Front Neurol 11:965 10.3389/fneur.2020.00965 PMC7493627 32982954

[52] Wittayanakorn N, Wong GM, Teti SA, Linan-Martinez VD, Qamar H, Cohen NT, Gaillard WD, Oluigbo CO (2025) Outcomes of reoperation following failed laser ablation surgery for epilepsy in pediatric patients. J Neurosurg Pediatr 35(4):369–378 10.3171/2024.11.PEDS24410 39919288

[53] Woods SB, Shields LB, Kuruvilla A, Shetty M, Feygin YB, Aras S, Mutchnick IS, Ali I, Karakas C (2025) Magnetic resonance–guided laser interstitial thermal therapy for pediatric drug-resistant epilepsy: a pooled analysis and systematic review of the literature. J Neurosurg Pediatr 36(6):702–13 10.3171/2025.5.PEDS25121 40972023

[54] Yu JS, Meade SM, Zhao R et al (2025) Expedited chemoradiation after laser interstitial thermal therapy (LITT) is feasible and safe in patients with newly diagnosed glioblastoma. Neuro-Oncol Adv 7(1):vdaf038 10.1093/noajnl/vdaf038 PMC12019959 40276375

[55] Zeller S, Kaye J, Jumah F, Mantri SS, Mir J, Raju B, Danish SF (2021) Current applications and safety profile of laser interstitial thermal therapy in the pediatric population: a systematic review of the literature. J Neurosurg Pediatr 28(3):360–367 10.3171/2021.2.PEDS20721 34214984

